# Effects of Chewing Exerciser on the Recovery of Masticatory Function Recovery after Orthognathic Surgery: A Single-Center Randomized Clinical Trial, a Preliminary Study

**DOI:** 10.3390/medicina56090483

**Published:** 2020-09-22

**Authors:** Hoon Joo Yang, Ik Jae Kwon, Akram Abdo Almansoori, Yoojung Son, Bongju Kim, Soung-Min Kim, Jong-Ho Lee

**Affiliations:** 1 Department of Oral and Maxillofacial Surgery, School of Dentistry, Seoul National University, Seoul 03080, Korea; yanghoonjoo@snu.ac.kr (H.J.Y.); ijkwon@snu.ac.kr (I.J.K.); dentist22@gmail.com (A.A.A.); smin5@snu.ac.kr (S.-M.K.); 2 Orthognathic Surgery Center, Seoul National University Dental Hospital, Seoul 03080, Korea; 3 Clinical Translational Research Center for Dental Science, Seoul National University Dental Hospital, Seoul 03080, Korea; dbwjd4220@naver.com (Y.S.); bjkim016@gmail.com (B.K.)

**Keywords:** masticatory function, postoperative physiotherapy, chewing exerciser, orthognathic surgery, electromyography

## Abstract

*Background and Objectives*: The aim of this study was to evaluate the effects of the chewing exerciser (CE) on the functional recovery of the masticatory muscles after orthognathic surgery. *Material and Methods:* This randomized clinical trial was conducted in patients undergoing bimaxillary orthognathic surgery including bilateral sagittal split ramus osteotomy. Postoperative physiotherapy (PT) was performed for 3 weeks starting 3 weeks after the surgery. The patients were randomly divided into two groups: control (Con) (conventional PT) group and CE group (use of CE in addition to conventional PT). The masticatory function was evaluated based on three standards: bite force (BF), amount of mouth opening (MO), and surface electromyography (sEMG) of the anterior temporal muscle (TA), masseter muscle (MM), sternocleidomastoid muscle, and anterior belly of digastric muscle before, 3 weeks (before PT) and 6 weeks after the surgery (after PT). *Results:* Finally, 22 subjects participated in this study: 10 patients for Con group and 12 patients for CE group. In both groups, the BF, which was reduced significantly after the surgery, recovered after the PT similar to that before the surgery. In both groups, the MO was also significantly reduced by the surgery. However, it did not recover as much, as it was before the surgery after applying the PT. There was no difference in BF and MO between the two groups. All muscles did not show significant changes in sEMG by surgery and PT at both resting and clenching states. *Conclusion:* Applying CE as a PT after orthognathic surgery did not cause any harmful side effects. In both groups, the weakened muscle activity after orthognathic surgery (OGS) was adequately restored 6 weeks after the surgery. However, CE did not offer a statistically significant benefit to the masticatory function in the recovery process after OGS.

## 1. Introduction

Orthognathic surgery (OGS) with/without orthodontic treatment is a commonly widely used approach for correction of the adult’s skeletal maxillomandibular discrepancy [1]. Patients requiring skeletal malocclusion treatment also seek it for functional rehabilitation and esthetic improvement [2]. OGS of the mandible is commonly performed using a bilateral sagittal split ramus osteotomy (BSSRO) technique [3]. The BSSRO results in a detachment of the masticatory muscles and change in the muscles position attached to the mandible to some extent. Although it is not necessary to strip the entire masseter muscle off the mandibular ramus and angle, the medial pterygoid muscle and stylomandibular ligament should be fully stripped off [4]. If these structures are not stripped well, positioning of the distal mandibular segment can be disturbed, and the proximal segment can be rotated unfavorably [4]. For these reasons, patients who have undergone BSSRO have a reduced function of masticatory muscles immediately after surgery. In addition, many clinical complications were observed in the masticatory muscles following OGS, such as muscular atrophy, decreased muscular strength and extensibility, and decreased muscle mass [5,6].

Physiotherapy (PT) can improve muscle vascularity and increase muscle mass and strength [7]. Kawazoe et al. (1982) suggested that therapeutic exercise of the stomatognathic system is effective in improving the masticatory function in patients with progressive muscular dystrophy [8]. In addition, maximum bite force (BF) can be easily increased with training in healthy young adult subjects [9]. Therefore, PT for functional recovery is required after OGS, which usually should be performed before the start of the postoperative orthodontic treatment that usually begins 3–6 weeks after the surgery.

PT performed after OGS varies depending on the operator, and passive exercise in the form of stretching by digital manipulation has been usually used [7,10]. Kato et al. (2012) reported that masticatory exercise using chewing gum after orthognathic surgery is effective for improving masticatory efficiency and maximal occlusal force in patients with jaw deformities [11]. However, the masticatory exercise began 6 months, postoperatively, and lasted for 3 months; therefore, it did not serve the purpose of obtaining functional rehabilitation before the postoperative orthodontic treatment. In addition, chewing gum was inadequate for patients undergoing orthodontic treatment with fixed appliances. 

**Figure 1:**
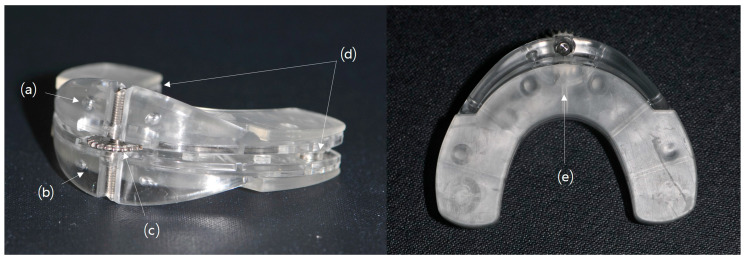
Chewing exerciser (NoSick, Hi-Feel world Co., Ltd., Seoul, Korea) for physiotherapy. (**a**) Upper part for maxillary teeth. (**b**) Lower part for mandibular teeth. (**c**) Height adjustment screw. (**d**) Elastic spring. (**e**) Silicon pad in both upper and lower parts, which is softened for indentation of teeth.

Chewing exerciser (CE) (NoSick, Hi-Feel world Co., Ltd., Seoul, Korea) is a masticatory exercise device that enables the isometric exercise of the masticatory muscle with springs attached to the posterior and anterior teeth areas (Figure 1). Using this device, the masticatory muscles can be exercised with the power of springs without disturbing by orthodontic brackets and wires.

The aim of this study was to evaluate the effects of the CE on the functional recovery of the masticatory muscles after OGS through assessment of bite force (BF), amount of mouth opening (MO), and surface electromyography (sEMG).

## 2. Materials and Methods

### 2.1. Study Design/Sample

**Figure 2:**
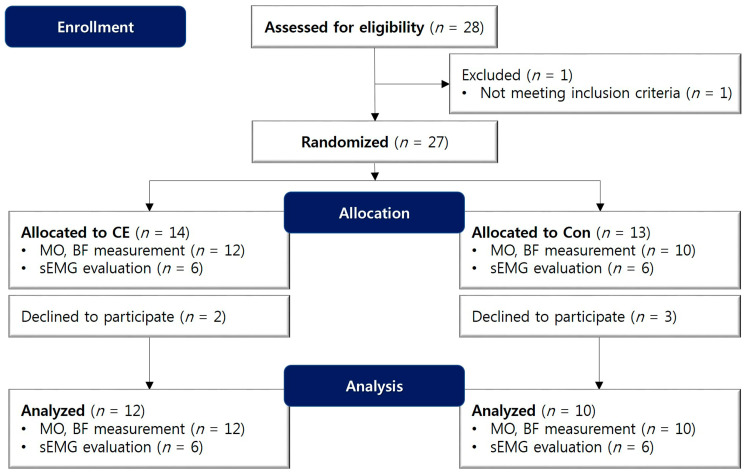
Flow diagram of a randomized clinical trial of the chewing exerciser as a tool of physiotherapy after orthognathic surgery. CE, chewing exerciser; BF, bite force; MO, amount of mouth opening; sEMG, surface electromyography.

**Figure 3:**
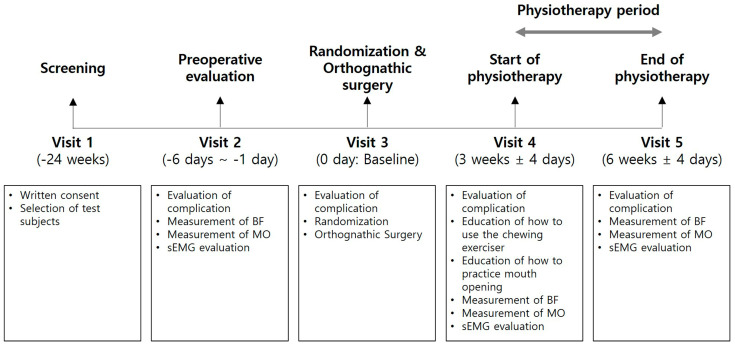
Clinical trial and evaluation content schemes. See Figure 2 for abbreviations.

This clinical trial was designed as a prospective, single-center randomized clinical trial. All subjects gave their informed consent for inclusion before they participated in the study. The study was conducted in accordance with the Declaration of Helsinki, and the protocol was approved by the Institutional Review Board of Seoul National University Dental Hospital from 12 December 2018 to 12 December 2019 (IRB No.: CDE17005) (Figure 2). Five-step study sequence (5 visits per subject) and timetable are listed in Figure 3.

To calculate the sample size needed for this clinical trial, the clinical study by Thombson et al. (2001) that provided maximum voluntary bite force changes after the isometric exercise was referenced [9]. The mean change in the BF after 3 weeks of exercise was 184.3 N, and its standard deviation was 174.0 N. If the sample sizes of both groups are the same, 12 subjects are needed for each group (*p* < 0.05, 1 − β = 0.8), and based on a 20% dropout rate, 14 subjects are required for each group (28 subjects in total).

The research nurse performed block randomization and allocated the subjects into two groups. Sequentially numbered, sealed, and opaque envelopes were prepared and provided to the subject to conceal the allocation at visit 4.

The subjects were skeletal malocclusion patients who visited the department of oral and maxillofacial surgery of Seoul National University Dental Hospital to undergo orthognathic surgery. All individuals signed an informed consent form and a registration number was given to each subject.

The inclusion criteria were healthy patients with skeletal malocclusion, older than 16 years, underwent preoperative orthodontic treatment, planned for Le Fort I osteotomy of the maxilla and BSSRO of the mandible as orthognathic surgery, with absence of pain in the TMJ area before surgery, and not diagnosed with temporomandibular disorder. The exclusion criteria were TMJ symptoms such as pain and/or noise, masticatory muscle pain or related diseases, the use of medications that may affect the masticatory muscle recovery, such as muscle relaxants, systemic diseases that may affect postoperative infection and postoperative recovery, and the need for additional surgery, such as mandibular angle reduction, which may affect the masticatory muscle attachment during orthognathic surgery.

### 2.2. Surgical Phase

Surgeries were performed by one oral and maxillofacial surgeon (HJY). All patients underwent conventional bimaxillary surgery. Le Fort I osteotomy was fixed using four miniplates at the piriform aperture and zygomatic buttress areas. Modified BSSRO was performed with the short-lingual technique [12]. The attachment of the masseter muscle was preserved as much as possible without stripping, and the medial pterygoid muscle was fully detached from the proximal segment. After removal of the intersegmental bone interference, the proximal segment was positioned at the most appropriate location in the anterior–superior direction with respect to the glenoid fossa. One four-hole mini-plate and four mono-cortical screws were used to fix the bone segments on each side. Because of the lack of bone contract between the proximal and distal segments in nine patients, a three-hole mid-plate was added to the external oblique ridge of the ascending ramus for stability. 

### 2.3. Use of Chewing Exerciser and Physiotherapy after Surgery

The subjects were randomly divided into experimental (CE) and control (Con) groups on the operation day (Visit 3), with allocation ratio of 1:1. Except that the CE was additionally used in the CE group, the remaining postoperative PT was similar in both groups. 

All patients were allowed a soft fluid diet for 10 days after surgery and then soft bland diet from day 10 to 3 weeks postoperatively. During this period, the final splint was applied at all times except the meal time, and loose intermaxillary fixation was performed using two orthodontic elastics. Afterwards, the strength of food gradually increased, and after 6 weeks, normal regular diet except for very hard and tough food was allowed. From about 10 days after the operation, it was recommended to put a warm steamed towel on the patient’s face according to the patient’s symptoms until the swelling was completely resolved. PT, including the mouth opening exercise (MOE), began around 3 weeks and continued until normal mouth opening (distance between upper and lower incisors tip >40 mm) was restored. After removing the elastics for intermaxillary fixation and the final splint, the MOE was performed using a thumb and an index finger for maximum mouth opening, maximum protrusion, and lateral excursion constantly for 5–10 min. Immediately after the exercise, the final splint was applied to check the displacement of the mandible. Then, the patient had a rest with the final splint application for at least 30 min. Securing the normal mouth opening was completed within 2–3 weeks in most patients.

During the recovery period, the CE group used the CE for 3 weeks from Visit 4 (3 week ± 4 day) to Visit 5 (6 week ± 4 day). After the CE’s silicone pad was softened by soaking in water at 70 °C for about 40 s, the patient was instructed to bite on it while being in the centric occlusion. After removing it from the mouth, it was soaked in cold water to fix the occlusal surface shape, and then the patient was informed how to use it. The mouth closing exercise using CE was performed by chewing the device 200 times in the morning, noon, and evening. Immediately after the exercise, occlusion checks using the final splint and breaks with the final splint application for at least 30 min were conducted. The CE was stored in a dedicated storage container when not in use.

### 2.4. Evaluation of Masticatory Function

The masticatory function was evaluated based on three parameters: BF, MO, and sEMG. Each examination was conducted at Visit 2, 4, and 5.

The BF was measured using a BF measuring device (Occlusal force-meter GM10, Nagano Keiki Co., Ltd., Tokyo, Japan). The Frankfort horizontal plane of the patient was made parallel with the ground. The measuring device was positioned on the first mandibular molar, and the patient was instructed to bite 3 times repeatedly to obtain the average value [13]. The same procedure was performed on the opposite side, and the average value was used for analysis. Using a metal ruler, the MO was measured as the distance between the upper and lower incisor tips during an assisted maximum mouth opening.

**Figure 4:**
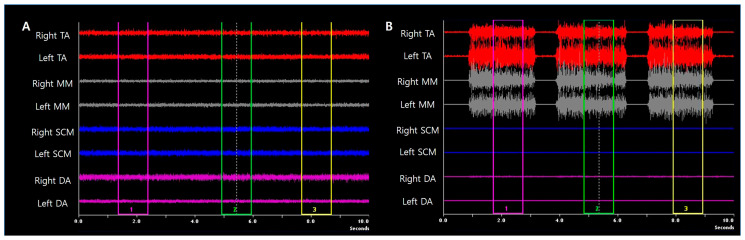
Analysis of surface electromyograph. (**A**). Waveforms during resting. (**B**). Waveforms during clenching. The average value of three original time domain waveforms (purple, green, yellow boxes) was calculated. The mean value of both sides was used for analysis. TA, anterior temporal muscle; MM, masseter muscle; SCM, sternocleidomastoid muscle; DA, anterior belly of digastric muscle.

The sEMG activity was recorded using a commercially available system for signal amplification and analog-to-digital conversion (BioEMG II, Bioresearch Assoc., Milwaukee, WI, USA). For the sEMG, the subjects were placed in a comfortable vertical sitting position without a headrest. The overlying skin of each muscle was wiped using an alcohol swab before attaching the electrode. The electrodes were placed over the muscle bulk and directed parallel to the general fibers. Electrical activities of a mandibular elevator/retractor muscle (anterior temporal muscle, TA), a mandibular elevator/protrusion muscle (masseter muscle, MM), neck extensor/protrusion/rotator muscle (sternocleidomastoid muscle, SCM), and mandibular depressor muscle (anterior belly of digastric muscle, DA) were evaluated. In monitoring the MM, the electrode axis was parallel to the muscle’s superficial fibers. On the TA muscle, the electrode axis was orientated with respect to the anterior border of the muscle and parallel to the fibers. With respect to the DA, the electrode was located below the lower border of the mandible, directed towards the hyoid bone. The electrode within the SCM was placed in half-length of the muscle, parallel to the fibers. The ground electrode was attached to the skin above the right clavicle. Muscle activity was recorded during resting and clenching state. The average value of three original times domain waveforms in each muscle was calculated, and the mean value of both sides was used for analysis (Figure 4).

### 2.5. Statistical Analysis

All data were anonymized and stored with the registration number given to the subject (Supplementary Materials). The measurements obtained were statistically analyzed using SPSS software, version 25.0 (IBM Inc., Chicago, IL, USA). The difference between Visit 4 and Visit 5 of BF and MO was represented by ∆BF and ∆MO, respectively. The Kolmogorov–Smirnov test was performed to determine whether the data had a normal distribution. The Mann–Whitney U test was used to analyze the differences between CE and Con groups to evaluate the effectiveness of CE use. Differences were considered to be statistically significant at *p* < 0.05. The alterations of BF, MO, and sEMG in each test at Visit 2, Visit 4, and Visit 5 (Visit 2 vs. Visit 4, Visit 2 vs. Visit 5, Visit 4 vs. Visit 5) were compared with Wilcoxon signed rank test with the Bonferroni correction to evaluate the effect of surgery and PT in each group. Statistical significance was defined as *p* < 0.0167.

## 3. Results

### 3.1. Subjects

Initially a total of 28 subjects (21.8 ± 3.6 years, M:F = 14:14) were enrolled in this clinical trial. After excluding 1 patient who did not meet the inclusion criteria, twenty-seven subjects remained (14 for CE, 13 for Con). Twenty-two of 27 subjects (78.57%) completed the study, and five subjects (17.86%; 2 for CE, 3 for Con) dropped from the study because of patients’ withdrawal or the need for additional mandibular surgery, such as condylectomy and mandibular angle reduction. Finally, 12 patients (22.3 ± 4.3 years, M:F = 7:5) were in the CE group comprised, and 10 patients (21.9 ± 2.9 years, M:F = 5:5) were in the Con group. The BF and MO were measured in all 22 patients, and sEMG was only completed in 12 patients.

Although there was an individual difference in the recovery period after the PT, all of the 22 patients restored the normal maximum opening, and no patient complained of pain or discomfort in the TMJ or surrounding muscles. In addition, no patient was found with instability of occlusion during the PT, and the final splint could be worn without a discomfort until referred for a postoperative orthodontic treatment.

### 3.2. Bite Force

In the CE group, the BF significantly decreased from 188.4 ± 134.0 kN before the surgery to 75.6 ± 33.4 kN at 3 weeks after the surgery (Visit 4) (*p* = 0.002). The BF recovered to 133.8 ± 55.1 kN after 3 weeks of PT (Visit 5), which was a statistically significant recovery (*p* = 0.003). The CE group showed no significant recovery in the BF after 3 weeks of the surgery. Similar findings were found in the Con group. The BF significantly decreased to 85.2 ± 28.3 kN in Visit 4 (*p* = 0.005) and significantly recovered to 129.6 ± 49.6 kN in Visit 5 (*p* = 0.007). The Con group also showed BF recovery without significant difference from before surgery at Visit 4. The BFs at Visit 2, 4, and 5 did not show a significant difference between the two groups (Table 1). 

### 3.3. Amount of Mouth Opening

In the CE group, the MO significantly decreased to 23.0 ± 6.7 mm at Visit 4 (*p* = 0.002) and significantly increased to 38.1 ± 6.7 mm at Visit 5 (*p* = 0.002). However, even when PT was performed for 3 weeks, it did not show as much MO as before the surgery. Even in the Con group, the MO decreased significantly to 27.5 ± 4.2 mm in Visit 4 (*p* = 0.005) and significantly recovered to 42.0 ± 3.9 mm in Visit 5 after 3 weeks of MOE (*p* = 0.005). The MO of the Con group at Visit 5 did not recover as much as before the surgery either (Table 2).

### 3.4. Surface Electromyography of Masticatory Muscles

The electrical activities at resting and clenching states of the mandible elevator muscles—TA and MMM—the neck extensor and rotator muscle—SCM—and the mandibular depressor muscle—DA—were measured using sEMG. All muscles did not show significant changes in sEMG by surgery and PT at both resting and clenching states (Table 3).

## 4. Discussion

In addition to the esthetic improvement, stable occlusion with restoring the function of the masticatory muscle is an essential goal of orthodontic surgery. Conventional PT, in which a mouth opening is performed using a finger force, has been performed after the surgery to restore the function of the masticatory muscles that were weakened by the surgery. The use of CE, a device that can strengthen a masticatory function as part of PT after surgery, was used in this prospective randomized study. The effects of the CE on the functional recovery of the masticatory muscles after OGS were evaluated using BF, MO, and sEMG assessments.

After the orthognathic surgery, three-dimensional changes of the jawbones and the surrounding soft tissue occur. The BF is measured with the occluding state of the first molar area, which is the region that exhibits the largest biting force on the occlusal surface [13]. In addition, the BF can be expressed as a vector sum of the tension force of all closed mouth muscles [13]. Therefore, alteration of occlusal plane angle and detachment of the masticatory muscle during OGS may change the BF [13]. In addition, the patients may show a reduced level of masticatory muscle activity after OGS because of the trauma, intermaxillary fixation, and soft diet [14]. In this study, the BF showed a dramatic decrease in the muscle activity following OGS at 3 weeks after surgery (V2). These findings are similar to the results of Ko et al.’s (2013) [15] and Celakil et al.’s (2017) [16] studies. After 3 weeks of PT, the BF recovered similarly to preoperatively about 6 weeks after surgery after PT in both groups.

MO is one of the most important indexes of a good postoperative recovery, which represents a range of motion of the TMJ [17]. In the Research Diagnostic Criteria/temporomandibular disorder index (RDC/TMD), myofascial pain with mouth opening limitation was also classified as a category of TMD [18]. In this study, the MO decreased immediately after surgery in both groups and increased to normal range as PT including mouth opening exercise was performed between Visit 4 and Visit 5. However, the MO at Visit 5 was 38.1 ± 6.7 mm in the CE group and 42.0 ± 3.9 mm in the Con group, which is significantly less than the MO of 52–53 mm before surgery. It was reported that the recovery of MO continued afterwards, and that it was recovered as much as before surgery about 6 months after surgery [19].

In the present EMG data, the standard deviation of the mean in the measurements ranged widely. EMG data can be influenced by various factors, and they can be difficult to obtain statistically significant results because it varies from patient to patient. These factors include muscle length, muscle anatomy, electrode position, and contraction filaments characteristics [20,21]. In addition, the postoperative EMG variation is due to changes in the vector of muscle force and the position of muscle insertion, and an increased number of contact points [22]. To improve repeatability in these studies, it is important to check the protocol for the method of electrode position and to be carried out by skilled examiner, since the cause of most other errors cannot be controlled [15].

It was reported that the masticatory muscle activity decreases after orthognathic surgery [16,23]. This initial decrease in EMG activity might be caused by the type of postoperative inter-maxillary fixation used or the decrease in the patient’s ability to manipulate their muscles, which occurred 6 weeks after orthognathic surgery [24]. It was expected that the weakened muscle activity would be improved again with the PT using CE, however exercise for 3 weeks was not enough to restore the electrical activity. The time needed to restore the EMG activities after OGS should be considered. Eshghpour et al. reported that MM showed decreased EMG activity at 3 months and that this activity had recovered at 6 months after OGS [23]. Likewise, Trawitzki et al. reported in their subsequent studies that EMG activity during chewing increased 6 months [25] or 3 years [26] after OGS compared with the preoperative period. The EMG value obtained at resting represents the resting tonus of the muscle, and in healthy muscle, it must be kept constant lower than the EMG value during clenching [10]. In this study, the resting tonus of TA was not increased in the Con group that had undergone conventional PT, which is consistent with the previous study by Ko et al. (2015) [10]. On the other hand, electrical activity of TA during resting was significantly increased by PT using CE in the CE group. Therefore, it was confirmed that the reinforcement of masticatory muscle by CE was preceded by TA rather than MM when used shortly for 3 weeks. In SCM and DA, no change of sEMG by OGS was observed, meaning that OGS has little influence on the sEMG of SCM and DA, unlike mandible elevator muscles.

There were some limitations that this study was conducted on a small number of subjects, and that the follow-up period was insufficient to observe muscle recovery by PT. In addition, the skeletal patterns of the subjects before surgery were diverse, which could make individual differences. CE showed no statistically significant improvement in the recovery of masticatory muscle function for 6 weeks after OGS. However, since MO and BF tended to increase numerically more in the CE group, it is necessary to conduct elaborate clinical studies with a large number of subjects with consistent skeletal patterns to more clearly determine the effectiveness of CE as part of PT after OGS in the future.

## 5. Conclusions

From this prospective randomized clinical trial, applying CE as a PT after orthognathic surgery did not cause any harmful side effects. In both CE and Con group, the weakened muscle activity after OGS was adequately restored 6 weeks after surgery. However, CE did not offer a statistically significant benefit to the masticatory function in the recovery process after OGS.

## Figures and Tables

**Table 1:** Comparison of bite force between chewing exerciser group (CE) and control group (Con) at Visit 2, 4, and 5.

	V2	V4	V5	∆BF	*p* Value		
					V2 vs. V4	V2 vs. V5	V4 vs. V5
CE	188.4 ± 134.0	75.6 ± 33.4	133.8 ± 55.1	58.3 ± 35.4	0.002 *	0.272	0.003 *
Con	153.8 ± 54.9	85.2 ± 28.3	129.6 ± 49.6	44.5 ± 35.1	0.005 *	0.114	0.007 *
*p* value	0.947	0.262	0.869	0.391			

Unite of values: kN. *p* value of group comparison with Mann–Whitney U test. *p* value of period comparison with Wilcoxon signed rank test with the Bonferroni correction. * *p* < 0.0167. V2, Visit 2; V4, Visit 4; V5, Visit 5; ∆BF, difference between Visit 4 and Visit 5 of bite force; CE, chewing exerciser; Con, control.

**Table 2:** Comparison of amount of mouth opening between chewing exerciser group (CE) and control group (Con) at Visit 2, 4, and 5.

	V2	V4	V5	∆MO	*p* Value		
					V2 vs. V4	V2 vs. V5	V4 vs. V5
CE	52.8 ± 8.4	23.0 ± 6.7	38.1 ± 6.7	15.1 ± 6.2	0.002 *	0.005 *	0.002 *
Con	53.1 ± 7.4	27.5 ± 4.2	42.0 ± 3.9	14.5 ± 6.6	0.005 *	0.008 *	0.005 *
*p* value	0.766	0.097	0.146	0.895			

Unite of values: mm. *p* value of group comparison with Mann–Whitney U test. *p* value of period comparison with Wilcoxon signed rank test with the Bonferroni correction. * *p* < 0.0167. ∆MO, difference between Visit 4 and Visit 5 in amount of mouth opening. See Table 1 for other abbreviations.

**Table 3:** Comparison of surface electromyography between chewing exerciser group (CE) and control group (Con) at Visit 2, 4, and 5.

			V2	V4	V5	*p* Value		
						V2 vs. V4	V2 vs. V5	V4 vs. V5
TA	Resting	CE	2.28 ± 0.84	2.43 ± 0.51	3.19 ± 0.94	0.686	0.249	0.046
		Con	1.68 ± 0.83	2.37 ± 0.80	2.14 ± 1.22	0.173	0.345	0.249
		*p* value	0.200	0.810	0.078			
	Clenching	CE	46.69 ± 28.24	38.56 ± 19.96	36.95 ± 15.39	0.345	0.345	0.917
		Con	55.48 ± 18.14	41.96 ± 12.98	43.80 ± 17.15	0.249	0.345	0.917
		*p* value	0.749	0.873	0.423			
MM	Resting	CE	1.78 ± 0.81	1.52 ± 0.42	1.47 ± 0.62	0.225	0.138	0.465
		Con	1.25 ± 0.22	1.69 ± 0.59	1.65 ± 0.67	0.116	0.249	0.893
		*p* value	0.092	0.873	0.631			
	Clenching	CE	48.49 ± 39.84	24.40 ± 19.56	23.98 ± 16.68	0.116	0.075	0.917
		Con	60.43 ± 15.51	28.89 ± 18.38	35.57 ± 24.93	0.028	0.116	0.753
		*p* value	0.200	0.522	0.423			
SCM	Resting	CE	2.13 ± 0.69	1.98 ± 0.46	1.81 ± 0.37	0.344	0.293	0.600
		Con	1.76 ± 0.44	2.31 ± 0.72	1.87 ± 0.25	0.078	0.463	0.345
		*p* value	0.228	0.377	0.872			
	Clenching	CE	3.13 ± 1.52	3.58 ± 1.77	4.08 ± 2.85	0.463	0.753	1.000
		Con	3.42 ± 1.69	4.71 ± 1.88	2.25 ± 0.47	0.173	0.141	0.028
		*p* value	0.688	0.197	0.127			
DA	Resting	CE	1.59 ± 0.70	1.28 ± 0.24	1.43 ± 0.39	0.223	0.674	0.461
		Con	1.48 ± 0.64	1.58 ± 0.33	1.55 ± 0.27	0.753	0.462	0.674
		*p* value	0.744	0.199	0.259			
	Clenching	CE	1.91 ± 0.88	2.23 ± 1.07	2.89 ± 2.08	0.686	0.345	0.463
		Con	2.53 ± 1.74	2.21 ± 0.79	2.24 ± 0.60	0.752	0.600	0.753
		*p* value	0.936	1.000	0.936			

Unite of values: μV. *p* value of group comparison with Mann-Whitney U test. *p* value of period comparison with Wilcoxon signed rank test with the Bonferroni correction. TA, anterior temporal muscle; MM, masseter muscle; SCM, sternocleidomastoid muscle; DA, anterior belly of digastric muscle. See Table 1 for other abbreviations.

## Supplementary Materials

The following is available online at https://www.mdpi.com/1010-660X/56/9/483/s1, the raw data of BF, MO, sEMG.
